# Pattern of diarrheal diseases in Atwima Nwabiagya District-Ghana, 2009- 2013

**DOI:** 10.11604/pamj.supp.2016.25.1.6207

**Published:** 2016-10-01

**Authors:** Nana Yaw Peprah, Donne Kofi Ameme, Samuel Sackey, Kofi Mensah Nyarko, Akosua Gyasi, Edwin Afari

**Affiliations:** 1 Ghana Field Epidemiology and Laboratory Training Programme (GFELTP); 2 Ghana Health Service

**Keywords:** Health Information Management System II, DHIMS, Atwima Nwabiagya, diarrhea, C2-Method, Ghana

## Abstract

**Introduction:**

Diarrheal diseases remain one of the most important public health challenges worldwide. In 2011, Ghana recorded average annual diarrheal cases of 2,218 per 100,000 populations for children under-five with Ashanti region recording the third highest. In the Atwima Nwabiagya District, summary statistics are done without detailed analysis. We analyzed diarrheal surveillance data to determine its pattern and to develop threshold levels for the disease in Atwima Nwabiagya District in the Ashanti Region of Ghana.

**Methods:**

District level diarrheal morbidity data from January 2009 to December 2013 was extracted from District Health Information Management System II, cleaned and analyzed. Descriptive analysis was done and expressed as frequencies and relative frequencies. Description of the data was done in time, place and person. We calculated diarrhea threshold using the C2 method.

**Results:**

Overall, 51,131 cases were reported with 55.2% being females over the five year period. The highest episode of diarrhea by age-group occurred in children under-five during the study period. Changes in disease occurrence did not conform to a seasonal pattern. District analysis showed one outbreak whilst sub-district analysis revealed more than one outbreak.

**Conclusion:**

Diarrheal disease pattern did not show a seasonal trend. Only one outbreak was observed at district level but each sub-district, showed more than one outbreak. The highest number of episodes of diarrhea per year occurred in Children under- five. Data analysis should be done at lower levels to inform interventions. Interventions should be targeted towards children under-five years.

## Introduction

Diarrheal diseases remain one of the most important public health challenges worldwide, particularly in developing countries where they cause high morbidity and mortality. Globally an estimated 1.7 billion diarrheal diseases are reported every year with majority occurring in Africa [1, 2]. Diarrhea is the second leading cause of death in children under five years and is also responsible for killing about 760 000 children every year globally[1]. Most deaths from diarrhea occur among children less than two years of age living in Southern Asia and sub-Saharan Africa [2]. In developing countries, children under three years old experience on average three episodes of diarrhea every year. Each episode deprives the child of the nutrition necessary for growth leading to malnutrition [1]. In 2011, Ghana recorded an average annual diarrheal cases of 2,218 per 100,000 population for children under-five with Ashanti region recording the third highest of 2,646 per 100,000 population [3]. Among children under-fives the highest prevalence of 33% was recorded in 12months-23months age group [4]. Diarrhea has been prioritized by many nations and attracts a lot of global attention. The integrated Global Action Plan for the Prevention and Control of Pneumonia and Diarrhea (GAPPD) aims at ending preventable childhood deaths due to pneumonia and diarrhea by 2025 [5]. Analysis of health-related data has proven useful for planning targeted interventions [6–8] to meet the GAPPD target. In Ghana, data on diarrheal diseases is collected routinely through the District Health Information Management System II (DHIMS II) but in-depth analysis is not done particularly at the sub-district and district level. In the Atwima Nwabiagya District, summary statistics are done without detailed analysis and action threshold levels are also not set to help guide interventions. As a result, public health officials are denied useful information for monitoring, controlling and prevention of diarrheal diseases. We therefore analyzed diarrheal surveillance data to determine its pattern and threshold levels in Atwima Nwabiagya District in the Ashanti Region of Ghana.

## Methods

**Study design:** all cause diarrhea surveillance data reported by health facilities in the district from 2009 to 2013 was extracted DHIMS 11 and analysis of this secondary data was done. DHIMS II is a database of priority diseases reported by all health facilities in the districts. Variables on diarrheal diseases in the DHIMS II were age, sex and sub district.

**Study area:** the Atwima Nwabiagya District is one of the 27 districts in Ashanti Region of Ghana. The District has five sub districts namely; Abuakwa, Akropong, Asuofua, Barekese and Nkawie. There are 17 health facilities which include hospitals, clinics, and health centers. It is peri-urban town with Barekese and Owabi dams all in Barekese Sub district which supply portable water to Kumasi and its environs including AtwimaNwabiagya District. The major rainfall season is from March to July and minor season is between August and mid-November. From 2010 population and housing census, the estimated 2013 population in the district is 161425 with 13.4% being children under-five.

**Study period:** the study was undertaken from 6th June to 11th July 2014.

**Data collection, processing and analysis:** diarrheal surveillance data from January 2009 to December 2013 for Atwima Nwabiagya District was extracted from DHIMS II database and exported into Microsoft Excel 2010. Data was cleaned and descriptive analysis was done and expressed as frequencies and relative frequencies. Description of the data was done in time, place and person. The thresholds for diarrhea were calculated using the C2 threshold method [9, 10]. The C2 method is defined as the sum of the mean and three standard deviations for seven preceding monthly diarrhea cases, skipping two most recent months.

**Ethical issues:** permission was obtained from the Regional and District Health Directorate of the Ghana Health Service. Data was not linked to patient identification; therefore one is unable to identify who has reported with an episode of diarrheal disease.

## Results

A total of 51,131 cases of diarrhea were reported during the period, of which 28,233(55.2%) were females. Children under five years had the highest number of cases of diarrhea 19818(38.8%) and age group 70years or more had the least 2554(5.0%)(Table 1).

**Table 1:** Characteristics of diarrheal cases in Atwima Nwabiagya district, 2009-2013

Variables	2009 N (%)	2010 N (%)	2011 N (%)	2012 N (%)	2013 N (%)	Total N (%)
**Sex**						
male	3918(7.66)	4766(9.32)	4402(8.61)	5128(10.03)	4684(9.16)	22898(44.7)
female	4896(9.58)	5319(10.40)	6150(12.03)	6077(11.89)	5791(11.33)	28233(55.22)
**Age-group**						
<5years	2970(5.81)	4055(7.93)	4831(9.45)	4016(7.85)	3946(7.72)	19818(38.76)
5yrs-9yrs	865(1.69)	578(1.13)	870(1.70)	962(1.88)	988(1.93)	4263(8.34)
10yrs-14yrs	608(1.19)	668(1.31)	710(1.39)	753(1.47)	755(1.48)	3494(6.83)
15yrs-19yrs	1025(2.00)	917(1.79)	933(1.82)	1101(2.15)	1124(2.20)	5100(9.97)
20yrs-49yrs	2331(4.56)	2568(5.02)	2043(4.00)	2801(5.48)	2422(4.74)	12165(23.79)
50yrs-69yrs	571(1.12)	792(1.55)	790(1.55)	1001(1.96)	583(1.14)	3737(7.31)
70yrs+	444(0.87)	507(0.99)	375(0.73)	571(1.12)	657(1.28)	2554(5.00)
**Sub-district**						
Abuakwa	1493(2.92)	1775(3.47)	2835(5.54)	2964(5.80)	2863(5.60)	11930(23.33)
Nkawie	2993(5.85)	2863(5.60)	3048(5.96)	4066(7.95)	3512(6.87)	16482(32.23)
Barekese	1423(2.78)	1723(3.37)	1051(2.06)	1206(2.36)	588(1.15)	5991(11.72)
Asuofua	1419(2.78)	1628(3.18)	1536(3.00)	790(1.55)	1752(3.43)	7125(13.93)
Akropong	1486(2.91)	2096(4.10)	2082(4.07)	2179(4.26)	1760(3.44)	9603(18.78)

**Figure 1:**
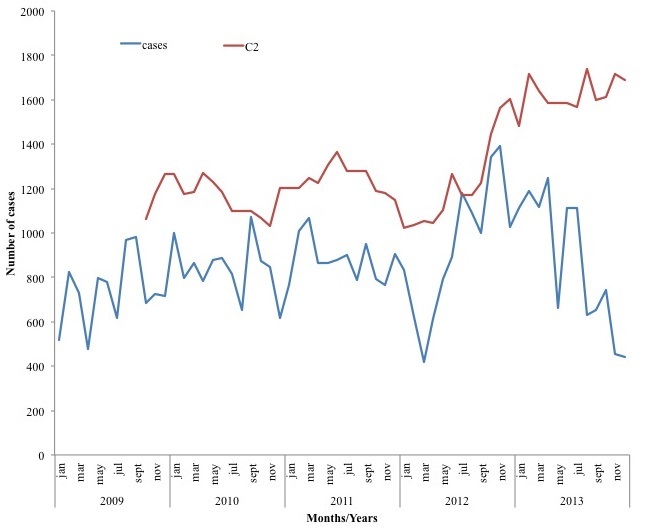
Trends of diarrheal cases with C2 threshold in AtwimaNwabiagya district from 2009-2013.

**Figure 2:**
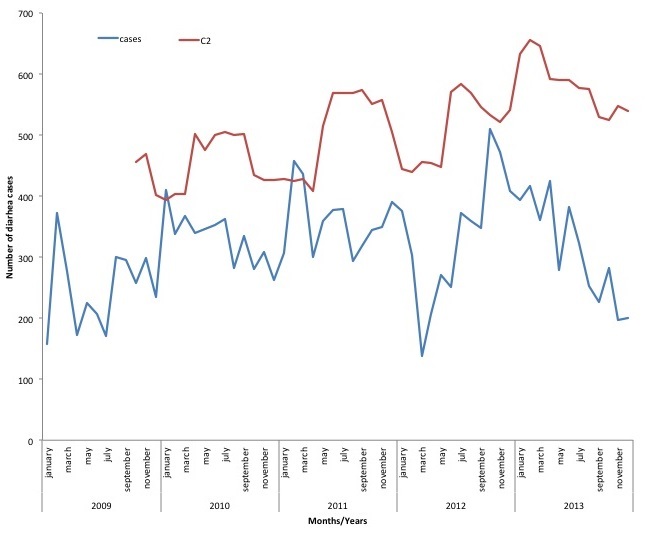
Trends of diarrheal cases and C2 threshold for children under-five years in Atwima Nwabiagya District from 2009-2013

**Figure 3:**
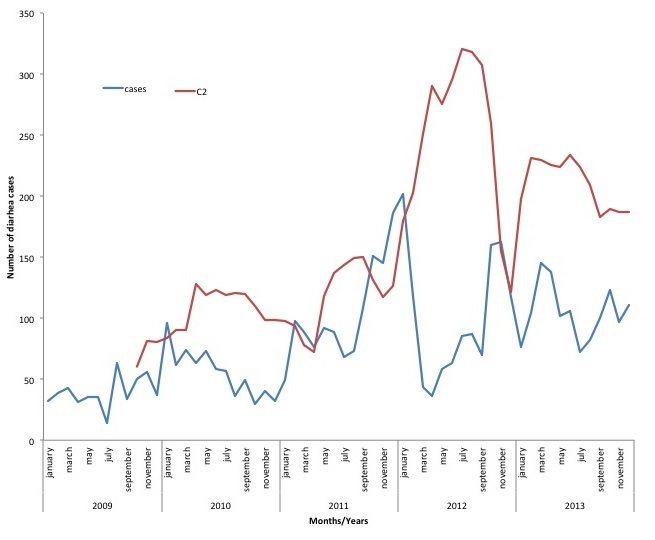
Trends of diarrheal cases and C2 threshold for children under-five years in Abuakwa sub-district from 2009-2013

The highest episodes of diarrhea per year occurred in children under-five years for all the years under review, whiles 10 -14years age group had the least number of diarrhea episodes for four years (2009, 2011, 2012, and 2013). In 2010, children 5 years - 9 years had the least number of episodes per age group per year. For the under-fives, those under a year old had the highest episode for 2011-2013 and one to four years age group recorded the highest for 2009-2010. The episodes of diarrheal diseases per thousand populations increased steeply from 2009 to 2010 then gradually to 2012 and then decreases in 2013. From January 2009, pattern was irregular with different peaking months per year. In 2009, Diarrheal diseases peaked in September (984) and had the lowest number of cases in April (476) with 2010 recording the highest in September (1073) and the lowest in December (617). In 2011 the highest number of cases were recorded in March (1068) and the lowest in January (768) whiles 2012 peaked in November (1391) and the lowest in March (418). Finally 2013 had it highest number of cases in April (1247) and in December (443) had the least number of cases. Data over the five years shows that, changes in disease occurrence do not conform to a regular seasonal pattern (Figure 1). Asuofua had the highest episodes of diarrhea per sub-district population in two different years (2009 and 2013), Barekese in 2010 and Nkawie (2011 and 2012). Abuakwa recorded the lowest in 2009 and 2010 whiles Barekese recorded the lowest in 2011 and 2013. In 2012, Asuofua recorded the lowest episode of diarrhea per sub-district. Using the C2 method, July 2012 recorded higher than the expected number of cases. This is the only month which recorded observed cases being more than the threshold (recorded 1182, expected-1171). For the sub district analysis, each sub district recorded more than the expected number. District level analysis for children under-five showed that January 2010, February-March 2011 had more reported cases than the expected (Figure 2) whiles sub-district for children under-five years revealed different multiple possible outbreak at different months and years. A typical example is shown in Figure 3.

## Discussion

This diarrheal surveillance data analysis was done to determine the pattern and to develop action threshold levels for the district. Few studies have used similar hospital data on diarrhea to define disease burden and trends in African countries [9–11]. Children under five years recorded the highest number of diarrhea episodes per year compared to the other age groups. This agrees with a research in Kenya that also revealed greater burden on children under five years than the other age groups [10] This may be because this age group include children who are crawling and put contaminated materials and fingers in their mouth. This may lead to a greater risk of having diarrhea and possible the reason why this age group had the highest in all the years. The episodes of diarrheal diseases per thousand populations increased steeply from 2009 to 2010 then gradually increased in 2012 and then finally decreased in 2013. The irregular increase and decrease agree by research [10] whiles others have recorded decrease over the years [12]. The increase may be as a result of the increase in health facilities over the period and the associated competition among health facilities which has improved quality of service by the various health facilities and therefore possibly attracting clients from adjacent districts. The decrease in 2013 may be as results of interventions put in place in 2012 by the district health management team, when the district had an alarming outbreak of cholera. The intervention included acceptable increase in chlorination of water by Ghana water and sewage, organizing and maintaining cleaning exercise. Sanitation task force increase surveillance and enforcement of sanitation laws This may have contributed to the decrease in the number of cases. Data over the five years shows that, changes in disease occurrence did not conform to a seasonal pattern which is the most reported form [10, 13]. In the district and sub-district analysis, the major peaks fell within and sometimes towards the end of the local rainy seasons of March to July and August to Mid-N November and this agrees with work done elsewhere [14, 15]. Sub-district having the highest rate of reported diarrhea varied from year to year and this findings agree with research [10]. Prevailing issues at a point in time may have influenced a particular sub district recording the highest prevalence of diarrheal diseases. Interventions to decrease diarrheal disease in some district may be better than others, so prevalence may vary among sub-district.

District analysis revealed only one month in which recorded cases were more than expected whiles sub-district analysis showed more than one over the five years. Sub-district analysis of children under-five revealed more months in which recorded cases were more than the expected and these occurred at different months and years compared to the district analysis. This means epidemiological patterns of diarrhea disease in the sub district may be different from the district picture and therefore the need to narrow down analysis to the lower level. Based on the findings we recommend to the District Health Management Team to do detailed sub district analysis to help them get clearer picture of what is happening at the lower level. This is important since aggregated data (district) may give a different picture compared to disaggregated data (sub-district). Interventions should be targeted at those under five and research should be conducted appreciate the reason for the trends seen.

## Conclusion

Overall, 51131 cases reported with 55.22% being females. The highest episode of diarrhea per age-group per year was recorded by children under-five. Changes in disease occurrence do not conform to a seasonal pattern. District analysis showed one outbreak whiles Sub district analysis revealed more than one outbreak.
